# Marginal Fit and Fracture Resistance of Vertical Versus Horizontal Margins in Monolithic Zirconia Crowns

**DOI:** 10.1002/cre2.70064

**Published:** 2025-01-23

**Authors:** Mohamed A. Salama, Mohamed F. Aldamaty, Moamen A. Abdalla, Elsayed Ali Omar, Mohammed H. AbdElaziz, Ahmed Yaseen Alqutaibi

**Affiliations:** ^1^ Fixed Prosthodontic Specialist Medical Services of the Egyptian Armed Forces Basrah Egypt; ^2^ Department of Fixed Prosthodontics, Faculty of Dental Medicine Al‐Azhar University Cairo Egypt; ^3^ Department of Restorative and Aesthetic Dentistry, College of Dentistry Almaaqal University Basrah Iraq; ^4^ Department of Substitutive Dental Science, College of Dentistry Imam Abdulrahman bin Faisal University Dammam Saudi Arabia; ^5^ Fixed Prosthodontics Department, Faculty of Dental Medicine Al‐Azhar University Cairo Egypt; ^6^ Department of Prosthodontics College of Dentistry, Taif University Taif Saudi Arabia; ^7^ Substitutive Dental Science, College of Dentistry, Taibah University Al Madinah Almonawarh Saudi Arabia; ^8^ Department of Prosthodontics Faculty of Dentistry, Ibb University Yemen

**Keywords:** fracture resistance, horizontal margins, marginal fit, monolithic zirconia, thermocycling, vertical margins

## Abstract

**Objective:**

The use of vertical margin design in all‐ceramic restoration has generated inquiries regarding its clinical efficacy under diverse dynamic oral conditions. This research aims to assess the marginal fit and fracture resistance of monolithic zirconia crowns featuring vertical margin design as opposed to those with conventional horizontal margin design.

**Materials and Methods:**

Two metal dies were employed to generate replicated resin dies mimicking mandibular first molar preparation. The metal dies were precision‐engineered with two margin designs: vertical margin design presenting a shoulderless configuration (Featheredge) and horizontal margin design (Radial Shoulder). Forty zirconia crowns were produced on the replicated resin dies using two varieties of monolithic zirconia, with twenty crowns in each category: pre‐shaded and multilayered zirconia. Both sets were further subdivided into two groups based on the finish line configuration utilized (n = 10). The vertical marginal gap of the zirconia crowns was gauged before and after thermocycling (5‐55oC/5000 cycles), followed by loading the crowns until fracture occurred. Statistical analysis was performed using one‐way analysis of variance (ANOVA), accompanied by Bonferroni's post hoc test and independent *t*‐test for pairwise comparisons.

**Results:**

The Shoulder subgroup of BruxZir exhibited the highest mean marginal gap value (120.06 ± 10.15 µ), while the Featheredge subgroup of BruxZir displayed the lowest value (49.72 ± 6.53 µ). Among the BruxZir group, the Featheredge subgroup showcased the highest mean fracture resistance value (4251.57 ± 279.90 N), whereas the Shoulder subgroup recorded the lowest value (1721.60 ± 225.16 N).

**Conclusion:**

Monolithic zirconia crowns with vertical margin design (Featheredge) demonstrated statistically enhanced performance compared to conventional horizontal margin design, as evidenced by lower marginal gap values and increased fracture resistance tolerance.

## 1 Introduction

The technologies and materials used in prosthetic and restorative dentistry have undergone significant advancements in recent decades, particularly in the development of biologically based aesthetic dental restorations (Amornvit et al. 2021). The flourishing digital era, complemented by integrated CAD/CAM technologies, has facilitated the improvement of traditional materials and the introduction of new ones. Digital technologies have simplified and expedited the fabrication of prostheses, making them more precise and reproducible. Zirconia, a machinable material, has played a crucial role in this progress by initially being employed to replace cast frameworks in fixed dental prostheses. Its exceptional mechanical properties, combined with the ability to mimic tooth shades despite its inherent opacity, have made it a popular choice. Zirconia also offers advantages such as radiopacity, reduced susceptibility to corrosion, superior chemical traits, dimensional stability, and an elastic modulus comparable to that of metal restorations (Denry and Kelly 2014).

Zirconia is a polymorphic substance that can exist in three distinct crystallographic forms – monoclinic, tetragonal, and cubic – depending on temperature and pressure conditions. In dentistry, the addition of 3% Yttria is commonly employed to stabilize the tetragonal phase in zirconia due to its superior strength, albeit at the cost of increased opacity necessitating subsequent porcelain veneering (Alraheam et al. 2019). The reduction of alumina content has been found to enhance the translucency of zirconia frameworks, leading to the development of translucent monolithic zirconia (3Y‐TZP) or second‐generation zirconia, which can function effectively without the need for additional veneering (Alqutaibi et al. 2022). Opting for a monolithic fabrication approach offers the advantage of streamlining the manufacturing process and minimizing the risk of chipping or delamination associated with a bi‐layer design (Kontonasaki, Giasimakopoulos, and Rigos 2020). Moreover, the conservative preparation guidelines associated with monolithic zirconia have been shown to help preserve hard dental structures and pulp integrity. Additionally, monolithic zirconia has been suggested to allow for the reuse of vertical margin design in such restorations (Poggio, Dosoli, and Ercoli 2012; Vigolo et al. 2015; Fuzzi et al. 2017).

The appropriate selection of margin design is crucial for achieving proper marginal fitting, which is closely associated with improved restoration fracture resistance (Holmes et al. 1989). In contrast, an inadequate marginal fit can result in significant marginal gaps that expose the luting cement to further dissolution, recurrent caries, and a risk of retention loss, ultimately leading to the failure of fixed dental restorations (Beuer et al. 2008; Souza et al. 2012; Eldamaty et al. 2020; Saab et al. 2018). Studies on ceramic restoration failures have shown a prevalence of cracks initiated at the margin, highlighting the importance of the relationship between fracture resistance and margin design (Sadeqi, Baig, and Al‐Shammari 2021; Lawson et al. 2019; Kim et al. 2012). The marginal fit of cemented restorations can be evaluated through non‐destructive direct magnification in either in‐vivo or in‐vitro settings to measure the resulting vertical marginal gap precisely (Holmes et al. 1989).

The impact of oral environment‐induced thermal aging on the durability of ceramic restorations during functional use has been a subject of interest (Sarıkaya and Hayran 2018; Yang et al. 2019). To study this, researchers often utilize simulated thermal fluctuation using a thermocycler as a reliable research methodology to mimic. This approach aims to replicate dynamic thermal stresses and analyze the effects of aging on fixed dental prostheses made with various materials and designs (Mitov et al. 2016; El‐Etreby, Metwally, and EL‐Nagar 2021; Borges et al. 2009). Different cycling protocols have been employed for thermocycling restorations, with reported cycling durations equivalent to more than 6 months of intraoral service (Mitov et al. 2016; Tekin and Hayran 2020). Thermal stresses have been observed to have a detrimental effect on the mechanical properties of translucent monolithic zirconia (3Y‐TZP), with the monoclinic transformation attributed to low‐temperature degradation, though lacking a definitive explanation (Mitov et al. 2016; Tekin and Hayran 2020). Conversely, the increased use of vertical margin design with this type of zirconia has raised concerns. This study seeks to explore the mechanical performance and marginal fitting of translucent monolithic zirconia (3Y‐TZP) with a vertical margin design. The null hypothesis posited is that the margin design will have no influence on the marginal fit or fracture resistance of monolithic zirconia crowns.

## 2 Materials and Methods

### 2.1 Study Design and Sample Size Calculations

**Figure 1:**
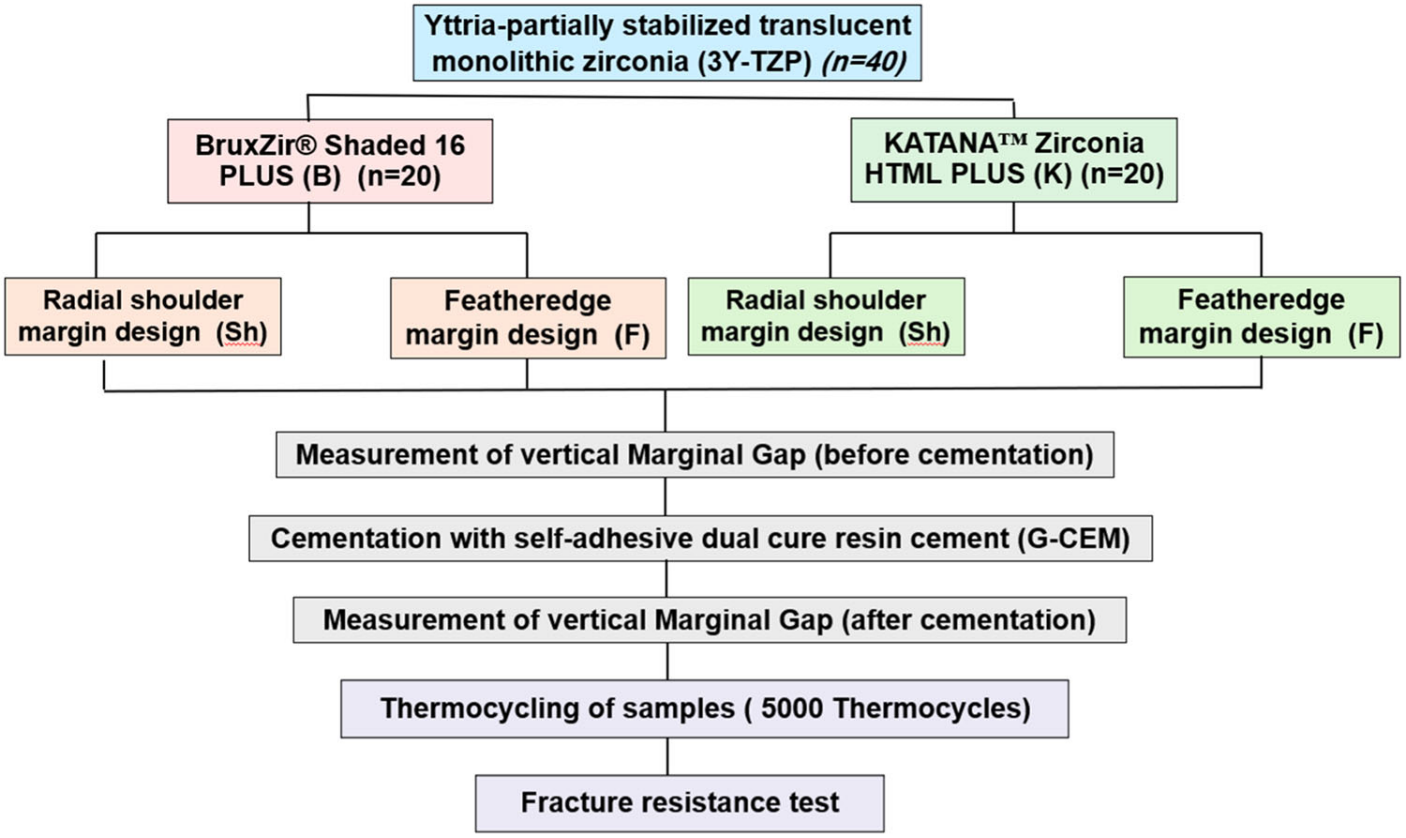
The progression of groups and methodologies implemented in this study.

This in vitro experimental study assessed two variations of monolithic zirconia, namely the pre‐shaded monolithic zirconia group (BruxZir Shaded 16 PLUS, Glidewell Laboratories, Irvine, USA), designated as Group B, and the multilayered monolithic zirconia group (KATANA Zirconia HTML PLUS, Kuraray Noritake Dental Inc., Japan), designated as Group K. Each of these groups (B and K) was further divided into two subgroups based on the finish line utilized, either Radial shoulder (Sh) or Featheredge (F), resulting in four subgroups (B‐Sh, B‐F, K‐Sh, and K‐F), as illustrated in Figure 1. This study did not involve human participants or animals, and therefore, ethics review and approval were not required.

The sample size calculation was based on prior research by Sadeqi et al. where the marginal gap ranged from 37.71 ± 11.73 to 39.49 ± 7.42, and the maximum load of fracture resistance ranged from 14,023 ± 2167 to 4,600 ± 618. The G*Power statistical power analysis program (version 3.1.9.4) determined a sample size of n = 10 for each subgroup, considering a large effect size (d) of 0.91 for marginal gap and 0.92 for fracture resistance, with an actual power (1‐β error) of 0.8 (80%) and a significance level (α error) of 0.05 (5%).

### 2.2 Experimental Design

**Figure 2:**
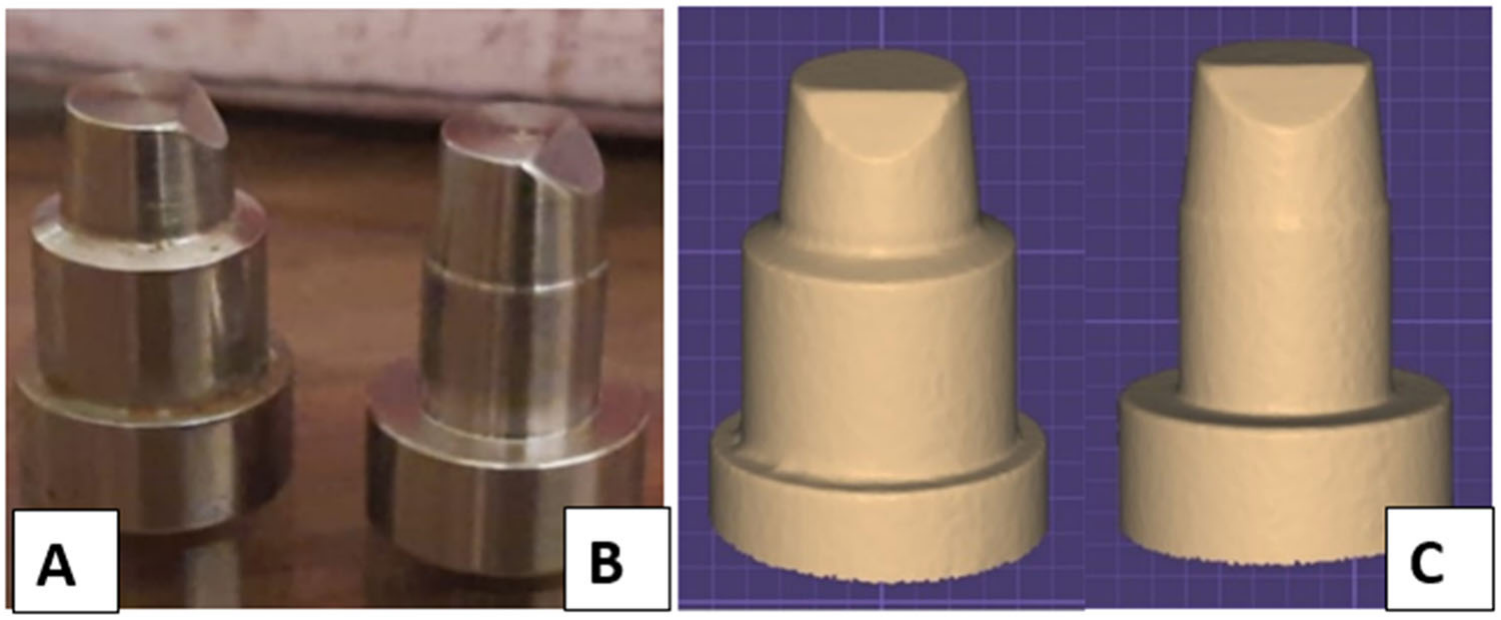
The metal dies used in this study. (A) Die with radial shoulder finish line. (B) Die with featheredge finish line. (C) Screen shoot for scanned models.

Two metal dies, shaped as circular truncated cones, were produced utilizing an industrial lathe milling device (Roland Corp., Los Angeles, CA, USA) to replicate the dimensions of a prepared lower first molar (Ezzat and Al‐Rafee 2020). The geometric features of the two metal dies included a flat occlusal surface, an occlusal bevel set at 45° (facilitating crown alignment and acting as an anti‐rotational element), an occluso‐gingival height of 5.0 mm, a convergence angle of 12°, an 8.0 mm wide base for stability, and different margin designs: one with a horizontal margin design consisting of a circumferential 1.0 mm radial shoulder (Sh) and the other with a vertical margin design featuring a 0.2 mm featheredge (F) finish line (Figure 2A and B) (Beuer et al. 2008). The metal dies were replicated to create the final epoxy resin dies. Duplicating silicone (Replisil 22 N, Silconic GmbH & Co. K – Germany) was utilized in a custom‐made tray, then filled with epoxy resin (Egypoxy, 10th of Ramadan city industrial zone, Egypt). Twenty duplicated resin dies were produced for each metal die (Souza et al. 2012; El‐Etreby, Metwally, and EL‐Nagar 2021). The epoxy resin was allowed to polymerize for a minimum of 24 h before being separated from their respective duplicating silicone mold (Figure 2C).

### 2.3 Specimen Preparation

**Figure 3:**
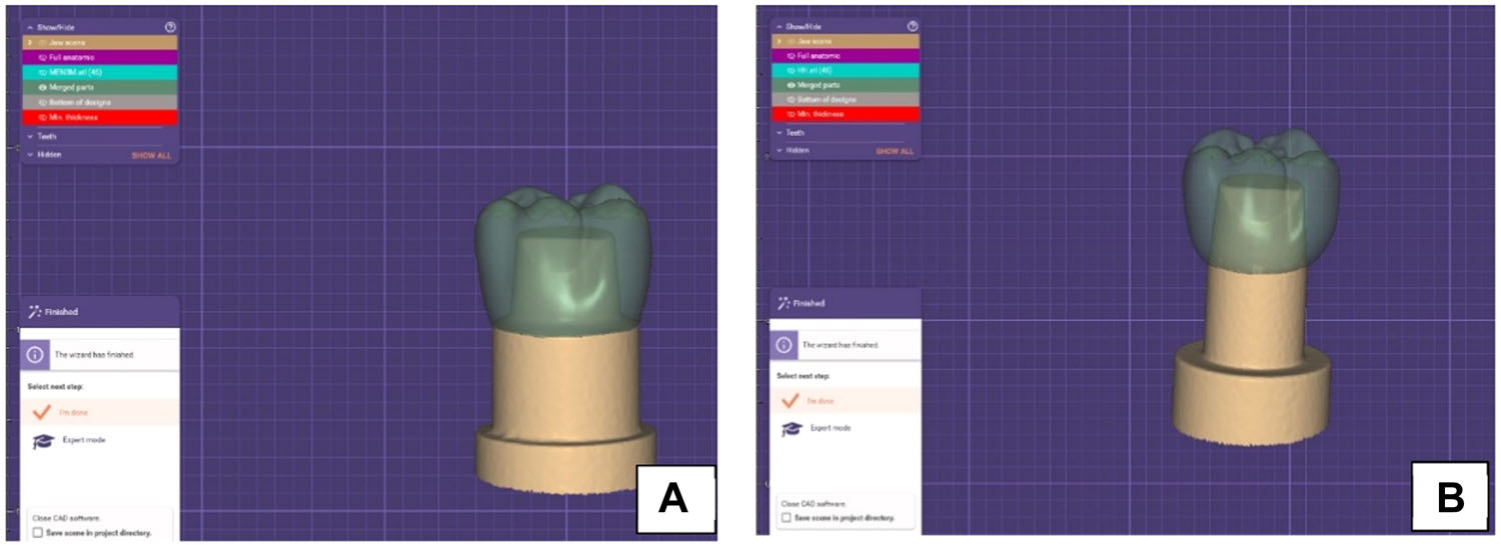
(A) Final design on the die with radial shoulder finish line. (B) Final design on the die with featheredge finish line.

An indirect laboratory scanner (Identica Hybrid, Medit, Seoul, Korea) was utilized to scan polymerized resin dies. Before each scanning process, the resin dies were treated with a scan spray (Shera scan spray, Shera GMBH, Lemförde, Germany). The scan data were then imported into CAD software (Exocad 3.0 Galway GmbH, Darmstadt, Germany) for the purpose of progressively designing the crown, with the cement space set at 50 µm (Souza et al. 2012) (Figure 3).

Crown‐shaped designs (n = 40 for the two groups) were transferred to a five‐axis milling machine (Roland DWX‐51D, Roland DGA Corp, California). The crown‐shaped specimens were sintered in a zirconia speed furnace (Nabertherm Speed, Nabertherm GmbH, Germany) following the manufacturer's instructions for BruxZir Shaded 16 PLUS (at 1580°C and a 150‐min holding time) and KATANA Zirconia HTML PLUS (at 1550°C and a 120‐min holding time).

**Figure 4:**
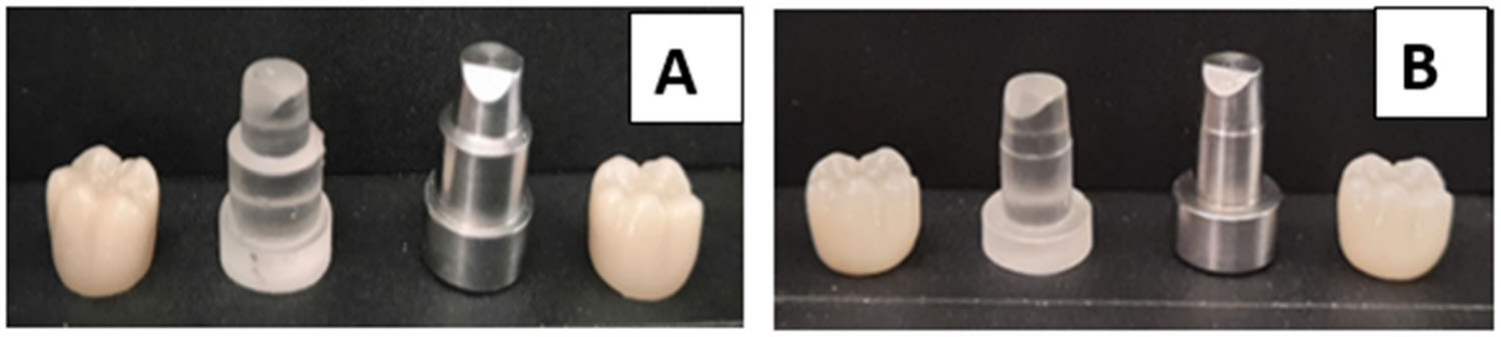
(A) The metal and resembling resin dies with radial shoulder finish line and corresponding crowns. (B) The metal and resembling resin dies with featheredge finish line and corresponding crowns.

**Figure 5:**
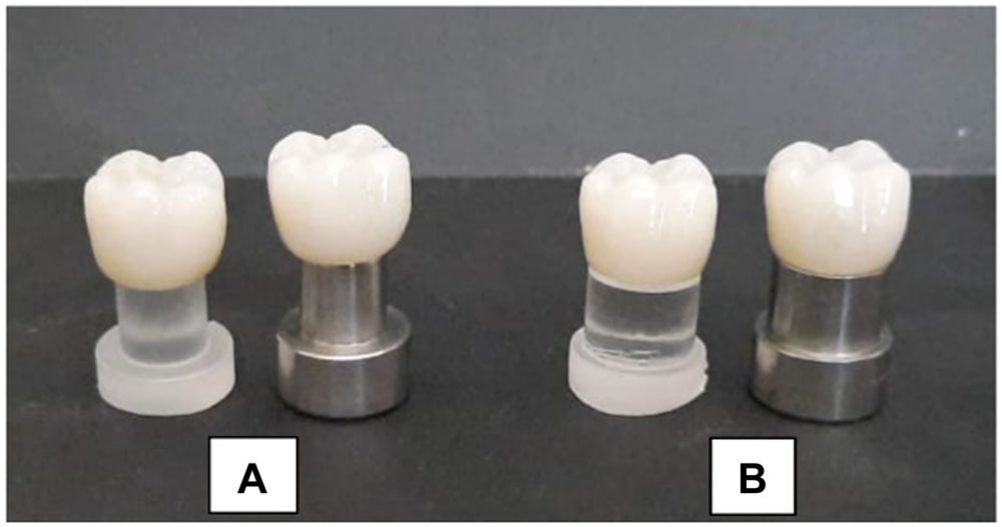
Zirconia crowns on their corresponding metal and resembling resin dies used in this study. (A) With featheredge finish line. (B) With radial shoulder finish line.

The intaglio surface of the sintered specimens was abraded using 50 μm alumina oxide (Al2O3) at a distance of 10 mm for 10 s (Lung and Matinlinna 2012). Crown‐shaped specimens were cemented with self‐adhesive dual‐cure resin cement (G‐CEM, GC America Corporation Inc, IL 60803). Initially, finger pressure was applied to seat the crowns on the corresponding dies; subsequently, a 4 kg vertical load was applied for 5 min. Excess cement was removed, followed by light curing for 20 s per surface to ensure a complete setting (El‐Etreby, Metwally, and EL‐Nagar 2021). Figures 4 and 5 depict zirconia crowns on their respective metal and resin dies used in this research.

### 2.4 Vertical Marginal Gap Measurement

**Figure 6:**
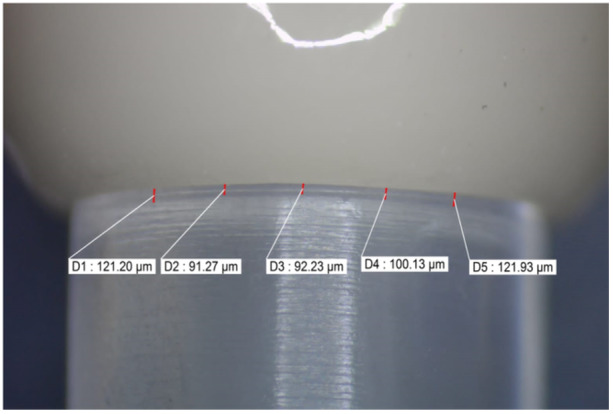
One of the shots taken and the measurement of the vertical marginal gap at the five landmarks.

The marginal fit was evaluated through the measurement of the vertical marginal gap both before and after cementation. The measurements were conducted using a stereomicroscope with a magnification power of X50 (Nikon‐SMZ645) equipped with a digital camera. A single image of each axial surface of the cemented crowns was captured. The analysis of these images was performed using the OmniMetTM Software (Buehler, Illinois, USA) for morphometric analysis at five equidistant points on each image, totaling 20 measurement points. The vertical marginal gap measurements were taken between the outer circumference of the die finish line (cavosurface angle) and the outer circumference of the crown margins following the methodology prescribed by (Holmes et al. 1989) All measurements were carried out by the same operator (Figure 6). Subsequently, the measurements were recorded and tabulated, and the mean vertical marginal gap for each subgroup was calculated.

### 2.5 Thermocycling Procedures

Thermocycling of cemented specimens was conducted using a Thermal cycler (THE‐1100, SD Mechatronik GMBH, Germany). A total of 5000 cycles were administered to all crown samples, which is equivalent to 6 months of intraoral service. The temperature fluctuation of the bath ranged between 5°C and 55°C, with a bath/dwell cycle of 60/10 s (Mitov et al. 2016; Tekin and Hayran 2020).

### 2.6 Fracture Resistance Test

The cemented specimens were subjected to static loading until failure using a universal testing device (Instron 3345, Instron Corp, MA, USA). A semi‐spherical steel head indenter with a diameter of 5 mm was positioned at the central fossa's midpoint on the occlusal surface of the crown. The loading was performed at a crosshead speed of 1.0 mm/min (Tekin and Hayran 2020). The maximum stress value required to fracture each specimen was measured in Newtons (N) by the device software (Blue Hill 3, Instron Corp, MA, USA). The recorded stress values were compiled in a table and the mean was calculated.

### 2.7 Statistical Analysis

The statistical analysis of all recorded measurements was conducted using SPSS (version 20.0, Armonk, NY: IBM Corp). The data were examined for normality through the verification of the data distribution with Shapiro–Wilk tests. In the case of vertical marginal gap, quantitative data were presented using mean and standard deviation. Paired *t*‐tests were utilized to assess the differences between vertical marginal gaps before and after cementation, while the Student *t*‐test was employed to compare the two groups under study. The significance level for the obtained results was set at 5%.

For the assessment of fracture resistance, the one‐way analysis of variance (ANOVA test) was employed to compare numerical variables with a normal distribution across groups. This was followed by Bonferroni's post hoc test and independent *t*‐test for pairwise comparisons. All P‐values were considered two‐sided, with statistical significance defined as *p* ≤ 0.05.

## 3 Results

### 3.1 Vertical Marginal Gap

The mean vertical marginal gap values were highest in the (B‐Sh) group (120.06 ± 10.15), followed by the (K‐Sh) group, and then the (K‐F) group, while the lowest mean value (49.72 ± 6.53) was observed in the BruxZir with featheredge (B‐F) group. Among the different zirconia groups, those with a radial shoulder finish line (Sh) exhibited significantly higher marginal gap values compared to those with featheredge finish lines (F) (*p* < 0.001). Pairwise comparisons revealed significant differences in finish lines within the same group (*p* < 0.001). The impact of cementation was notable for both materials across various finish line configurations, leading to larger marginal gap measurements. Nevertheless, the influence of cementation was not significant for both finish line types across materials. Specifically, when using an (F) finish line, the Katana and BruxZir groups did not exhibit a statistically significant difference after cementation (*p* = 0.289). Similarly, with an (Sh) finish line, the Katana and BruxZir groups also showed no significant difference after cementation (*p* = 0.976) (Table 1).

**Table 1:** Before cement and after cementation comparison according to vertical marginal gap (µm) in each material and each type of margin.

Ceramic material	Cementation	Vertical marginal gap (µm)		*p_1_*
		F	Sh	
Katana (K)	Before cement	46.75 ± 10.47	101.4 ± 16.23	< 0.001*
	After cement	53.70 ± 9.47	119.8 ± 19.90	< 0.001*
	*p_0_*	< 0.001*	< 0.001*	
Bruxzir (B)	Before cement	45.47 ± 5.83	97.88 ± 8.91	< 0.001*
	After cement	49.72 ± 6.53	120.1 ± 10.15	< 0.001*
	*p_0_*	< 0.001*	< 0.001*	
*p*_*2*_	Before cement	0.739	*0.619*	
	After cement	*0.289*	*0.976*	

*Note: p0: p* value for comparing between Before cement and After cement in each finish line separately. *p1: p* value for comparing between F and Sh in each group. *p2: p* value for comparing between Katana and Bruxzir in each group.

Abbreviations: B = Bruxzir, F = featheredge finish line, K = Katana, Sh = radial shoulder finish line.

* Significant, Significance level *p* ≤ 0.05.

### 3.2 Fracture Resistance

The mean fracture resistance values exhibited the greatest magnitude within the (B‐F) group at 4251.57 ± 279.90, whereas the lowest value was observed in the (B‐Sh) group at 1721.60 ± 225.16. Both featheredge groups demonstrated significantly higher fracture resistance values compared to the radial shoulder groups. In particular, the radial shoulder in the (K‐Sh) group displayed significantly higher fracture resistance than the (B‐Sh) group (*p* = 0.000) (see Table 2). Notably, the utilization of a featheredge finish line did not yield a statistically significant difference between the Katana and BruxZir groups.

**Table 2:** Descriptive statistics of maximum load of fracture resistance (*N*) in different groups (ANOVA test).

Groups	Mean	Std. Dev	95% Confidence Interval for Mean		Min	Max	*p* value
			*Lower Bound*	*Upper Bound*			
K‐F	3974.48^a^	419.83	3674.15	4274.81	3265.23	4439.75	0.000*
K‐Sh	2399.52^b^	261.24	2212.64	2586.40	2109.07	2910.59	
B‐F	4251.57^a^	279.90	4051.34	4451.79	3851.84	4687.63	
B‐Sh	1721.60^c^	225.16	1560.53	1882.67	1434.55	2056.76	

*Note:* Post hoc test: means sharing the same superscript letter are not significantly different.

Abbreviations: B = Bruxzir, F = featheredge finish line, K = Katana, Sh = radial shoulder finish line.

* Significant, Significance level *p* ≤ 0.05.

## 4 Discussion

In the current investigation, the focus was on the implementation of a conservative vertical margin design using translucent monolithic zirconia (3y‐TZP). As indicated by the results, the initial null hypothesis was rejected, demonstrating that the margin configuration had a statistically significant impact on both marginal fitting and fracture resistance. The primary goal of contemporary restorative dentistry is to repair damaged dentition, emphasizing minimally invasive strategies alongside advancements in ceramic materials. Conservative margin designs, specifically those below 0.5 mm, have been identified as indefinite finish lines or vertical preparations. Originally, these designs were introduced as feather or chisel finish lines in conjunction with cast restorations. Vertical preparations employing feather or chisel techniques are classified as shoulderless vertical preparations, while the edgeless category of vertical preparation relies on area preparation rather than delineated finish lines and has more recently been referred to as the biologically oriented preparation technique (BOPT). In our study, shoulderless vertical preparation was chosen due to its reduced tissue invasiveness and simplified production when compared with edgeless crown preparation, facilitating further comparison with conventional radial shoulder preparation (Łabno and Drobnik 2020).

To enhance the consistency and reliability of in vitro studies, the standardization of test specimens has been recommended. Although natural teeth have been commonly employed in such studies, a number of inconsistencies have been identified concerning parameters such as age, anatomy, size, form, and the duration of storage post‐extraction (Nawrocka and Łukomska‐Szymańska 2019). In the current investigation, two standardized stainless‐steel master dies were replicated to generate epoxy resin dies. Resin‐based dies have been implemented as substitutes for natural teeth in in vitro environments. The expectation was for these resin‐based dies to exhibit similar bulging characteristics as dentin does under induced mechanical loading conditions, thereby minimizing the variations observed among extracted natural teeth (Lawson et al. 2019; Łabno and Drobnik 2020).

The cement space thickness for all crown samples was standardized to 50 µm, as determined to offer optimal marginal fit based on findings from a prior study (Eldamaty et al. 2020). In adherence to the protocol advised in another study, each crown underwent air abrasion of its fitting surfaces using 50 μm Al_2_O_3_ for 10 s at a distance of 10 mm (Lung and Matinlinna 2012). The crowns were cemented to their corresponding epoxy resin dies using adhesive resin cement as follows before, to avoid bias induced by resin‐modified glass‐ionomer cement since previous studies have found that cement has a greater effect on retention and higher fracture load (Lawson et al. 2019).

The direct viewing technique is widely noted in the literature as the predominant method for measuring marginal discrepancies (Eldamaty et al. 2020). In this study, the vertical marginal gap was evaluated through direct observation under a stereomicroscope with a 50× magnification at 20 selected sites per crown. There have been apprehensions raised regarding the extended durability of zirconia‐based monolithic restorations in the intraoral environment, characterized by humidity, thermal variations, and mechanical stresses. The phenomenon of low‐temperature degradation has been linked to the monoclinic phase transformation, leading to a subsequent decline in mechanical properties (Yang et al. 2019).

Thermocycling is a valuable method used to assess the clinical efficacy of restoration by replicating the temperature fluctuations present in the oral cavity (El‐Etreby, Metwally, and EL‐Nagar 2021). By investigating the discrepancies in thermal and fatigue resistance among different materials, valuable insights can be gained into their clinical shortcomings (Sarıkaya and Hayran 2018). Despite the brevity of the aging period in the present study (6 months), it is noteworthy that previous research has shown that zirconia can manifest thermal damage and related alterations within relatively short timeframes (6–12 months), thus inducing significant thermal aging conditions (Yang et al. 2019). The fracture resistance was assessed post thermocycling, as Tekin et al. posited that evaluating the material's strength after aging could help elucidate the exact performance of dental materials, mirroring conditions found in authentic oral environments (Tekin and Hayran 2020).

Generally, the impact of the finish line design on the marginal fit was presenting an argument, some were in agreement (Souza et al. 2012), while opposed by others (Vigolo et al. 2015; Eldamaty et al. 2020). Further investigations focused on the finish line design have recommended using the shoulder finish line since a smaller marginal discrepancy compared to vertical marginal design types was evidenced (Souza et al. 2012). Such a conventional horizontal margin design was advocated and implemented extensively just before the recent introduction of high‐strength ceramic materials (McDonald 2001). These recommendations were not in agreement with the clinical investigation of Poggio, Dosoli, and Ercoli (2012) as they revealed that vertical margin design allows a clinical performance like the other margin designs' reports but using less invasive preparations.

There was a growing body of scientific evidence supporting the validity of vertical preparations (Valenti and Valenti 2015). The results of the present study were in line with the previous clinical study (Poggio, Dosoli, and Ercoli 2012) revealing significant reduction of the resultant marginal gap with featheredge finish line as a shoulderless type of vertical margin design. The marginal gap values were around 50 microns in both zirconia groups with feather design while the shoulder preparation hits near the upper limit of reported marginal reaching about 100 microns. This improvement with vertical preparation fitting may be attributed to the new fabrication technology that rendered the fabrication of thin‐edged ceramic restoration more reliable (Denry and Kelly 2014), along with the improvements in mechanical properties of the materials (Camposilvan et al. 2018). Additionally, in a clinical context, precise fine preparations were achieved through the use of high magnification, either with loops or in‐office mounted microscopy (Eichenberger et al. 2018).

From a clinical perspective, vertical margin design is recommended when monolithic restorations are desired and less invasive preparation is necessary. This conservative approach is advantageous in various clinical scenarios, such as cases involving teeth with endodontic treatment, the vital teeth of young individuals, and dental irregularities at the gingival third of the teeth (Vigolo et al. 2015). Despite notable variability in marginal gap values between the two types of finish lines employed in the study, the maximum recorded values remained within the clinically acceptable range, which is below 150 μm (Holmes et al. 1989).

Regarding the resulting fracture resistance values, there was no clear distinction observed among the tested margin designs. It was noted that shoulder finish lines exhibited higher fracture resistance compared to thinner or vertical finish line types (Kim et al. 2012). These findings align with previous studies, which also reported elevated fracture resistance values with featheredge preparations (Beuer et al. 2008; Mitov et al. 2016).

The results of the current study demonstrate a substantial increase in fracture resistance values, nearly double the strength observed in the radial shoulder configuration. The enhanced values associated with featheredge preparation can be attributed to a more favorable stress distribution under loading conditions. This preparation allows for a slight apical sliding without a definite stop when subjected to excessive loading, thereby limiting further marginal stress concentration. It is crucial to consider adequate occlusal thickness to manage the increased stresses generated occlusally (Saker et al. 2024). An influential factor affecting fracture resistance testing is the condition of cementation, with resin cementation and thin ceramic margins proving to be pivotal. Previous studies have shown that conventional cementation using glass ionomer cement resulted in poor performance, particularly following thermocycling (Luthy, Loeffel, and Hammerle 2006). Resin cementation facilitates improved stress distribution and enhances the overall structural resistance (Lawson et al. 2019; Luthy, Loeffel, and Hammerle 2006).

The physiologically posterior region exhibits an average occlusal force of approximately 700 N and a maximum occlusal force of 1000 N (Altayyar et al. 2023). Consequently, all findings concerning the fracture resistance of the crown samples significantly surpass the maximum occlusal force (1000 N) and are deemed clinically acceptable.

From a materialistic viewpoint, two variations of monolithic zirconia with heightened translucency were evaluated: pre‐shaded and multilayered monolithic zirconia. Research findings demonstrated that the nature of zirconia influences their adaptation (Saab et al. 2018) and resistance to fracture (Lawson et al. 2019). The outcomes of this particular study substantiate the relationship between fracture resistance and the type of monolithic zirconia analyzed. Specifically, the fracture resistance of multilayered monolithic zirconia exhibited statistically superior performance compared to the pre‐shaded variant with a radial shoulder finish line, potentially attributable to the applied materials fabrication technology and methodology (Kontonasaki, Giasimakopoulos, and Rigos 2020).

The results of the current study provide strong support for the implementation of vertical margin designs, such as featheredge, in combination with translucent monolithic zirconia (3Y‐TZP) crowns. This approach allows for the realization of a minimally invasive concept while also ensuring mechanically robust restorations with satisfactory esthetic outcomes.

Further future testing incorporating dynamic loading over extended periods and fractographic analysis could provide additional insights into the mechanical behavior of monolithic zirconia restorations.

## 5 Conclusion

With the limitations of this study, we conclude that

1. The vertical finish line (featheredge) presents a better fit and enhanced fracture resistance compared to the radial shoulder finish line, albeit both are deemed clinically acceptable.
2. When considering radial shoulder designs, it is found that multilayered zirconia exhibits elevated strength relative to pre‐shaded zirconia.
3. Both types of finish lines surpass the specified clinical fracture resistance standards, with the vertical finish line being suggested for minimally invasive tooth preparation in conjunction with translucent monolithic zirconia crowns.

## Author Contributions

**Mohamed A. Salama, Mohamed F. Aldamaty,** and **Moamen A. Abdalla:** conceptualization, methodology, investigation, results, writing e‐original draft, writing e‐review and editing, project administration, supervision. **Elsayed Ali Omar** and **Mohammed H. AbdElaziz:** conceptualization, methodology, investigation, results, writing e‐original draft, writing e‐review, and editing. **Ahmed Yaseen Alqutaibi:** results, writing e‐original draft, writing e‐review, supervision.

## Conflicts of Interest

The authors declare no conflicts of interest.

## Data Availability

The data that support the findings of this study are available from the corresponding author upon reasonable request.
